# Necrology

**Published:** 1898-03

**Authors:** 


### NECROLOGY.

Charles Ernest Cornevin was born at Issen-Bassigny, France, in 1846, and died November 24, 1897. The deceased, after graduating from the Lyons Veterinary School, France, was elected assistant to M. Saint-Cyr, of the Lyons Faculty, who recognized his early ability while yet a student. He retired to general practice in 1868, edited the *Annales de Zoötechnie et de Médécine Vétérinaire*, one of the first journals published outside of the veterinary schools. His early investigations pertained to questions belonging to zoötechny, and in 1876 he was elected to that chair in the Lyons Veterinary School, which he held at the time of his death. He was also professor in the School of Agriculture at Ecully, and was one of the editors of the *Journal de Médécine Vétérinaire et de Zoötechnie.*

M. Cornevin's publications are numerous. Among his works published are the following: *Du Lemodex Canins; Le Charbon Symptomatique; Des Résidus Industriels dans l'Alimentation du Bétail; Production du Lait; Traité de l'Age des Animaux Domestiques d'Apres les Dents et les Productions Epidermiques; Les Oiseaux de Basse-cour; Les Petits Mammiféres de la Basse-cour et de Maison; Traité de Zootechnie Générale; Traité de Zootechnie Spéciale.*

The last two works stand above all others as general text-books. To the above list must be added numberless other articles printed in periodicals, especially on anthrax and symptomatic anthrax, on subjects pertaining to veterinary science, and particularly zoötechny. His writings are based on original investigations and upon observations made upon animals on the experimental farm owned by the Lyons school, and directed by the chair of zoötechny.

The deceased was a member of many professional and honorary societies, and was held in the highest respect by his colleagues and other associates. The veterinary profession, both in Europe and in America, where his writings are well known, sincerely regrets the loss of one of its able members and most voluminous writers. He was truly one of those of whom the profession should have many more.

S. J. J. HARGER.

### DAVID P. FRAME, M.D.C.

JUST as we are going to press with this issue the news reaches us of the sad and untimely death of our colleague, Dr. D. P. Frame, of Colorado Springs, Colorado. His death occurred at Kansas City, Kansas, just two days after his arrival there to accept the post of duty in the U. S. Meat-inspection Service, where he had been ordered to report for duty. Just previous to leaving Colorado he had been ill from Bright's disease, and the anxiety and extra labor in closing up his affairs and the removal of his family were followed by bronchial pneumonia and death on February 25th.

Dr. Frame was a genial, earnest, and progressive worker in the profession; a member of the U. S. V. M. A.; active in the work of his State Association, and in agitating the need of legislation in the "Boulder State." He had won the position of Food Inspector in the city where he lived, and was in close touch with all veterinary sanitary measures and board of health movements for his people.

He received his degree from the Chicago Veterinary College in 1894. Several years were spent in his education in special study and laboratory work, and he was well equipped in the work of food inspection.

The Order of Elks, of which he was a member, took charge of the funeral ceremonies, on the 28th, at Kansas City.

